# Designing synthetic consortia of *Trichoderma* strains that improve antagonistic activities against pathogens and cucumber seedling growth

**DOI:** 10.1186/s12934-022-01959-2

**Published:** 2022-11-11

**Authors:** Dazhi Hao, Bo Lang, Yongkun Wang, Xinhua Wang, Tong Liu, Jie Chen

**Affiliations:** 1 School of Agriculture and Biology, Shanghai Jiao Tong University, Shanghai, People’s Republic of China; 2 The State Key Laboratory of Microbial Metabolism, Shanghai Jiao Tong University, Shanghai, 200240 People’s Republic of China; 3 Key Laboratory of Green Prevention and Control of Tropical Plant Diseases and Pests, Ministry of Education, College of Plant Protection, Hainan University, Haikou, 570228 People’s Republic of China

**Keywords:** *Trichoderma*, Co-culture, Combination design, Consortia

## Abstract

**Background:**

*Trichoderma* spp. are important agricultural biocontrol microorganisms that are often used as effective components of microbial fungicides and microbial biofertilizers. However, most of these products are prepared by a single strain in monoculture, which significantly limits the biocontrol efficiency and stability of *Trichoderma* products. Therefore, the establishment of a design and screening approach for consortia with multi-*Trichoderma* strains for co-culture is of great importance to overcome the shortage of traditional *Trichoderma* biocontrol products.

**Results:**

First, 15 *Trichoderma* strains were screened in terms of mycelium growth rate, antagonistic activity to a variety of pathogens, stress tolerance to high temperature and salt stress, and cucumber seedling growth promotion level. Then, the combinations of *Trichoderma asperellum* GDSF1009 (CGMCC NO. 9512), *Trichoderma asperelloides* Z4-1 (CGMCC NO. 40245), *Trichoderma harzianum* 10569 (CGMCC NO. 40246), and *T. asperellum* 10264 (CGMCC NO. 22404) were finally screened as an optimal consortium for co-culture underlying the levels of plant growth-promoting and antagonistic activity to *Fusarium oxysporum* and seed germination promotion relative to the monoculture of a single strain. Consortia with multiple co-cultured strains were found to generate larger amounts of free amino acids than those from the monoculture of a single strain, and a pot assay also indicated that metabolites of co-cultures were able to promote cucumber seedling growth superior to that with monoculture of a single strain, even though the promotion was better than from simply mixed cultures from each of the four *Trichoderma* strains. Taken together, the co-culture consortia composed of the four compatible interactive *Trichoderma* strains was a potential novel multiple strain biocontrol agent based on the combination of synthetic consortia design and co-culture. In the field experiment, we found that the growth-promoting effect of the co-culture fermentation filtrate was better than that of the single culture fermentation filtrate. Compared with T-Z4-1, T-1009, T-10264 and T-10569, the plant height of cucumber was increased by 22.99%, 42.06%, 24.18% and 30.09%, respectively, and the stem diameter was increased by 16.59%, 18.83%, 13.65% and 14.70%, respectively.

**Conclusion:**

An approach to designing and screening *Trichoderma* consortia for co-culture was established. The consortia co-culture presented a better performance in antagonistic activity and cucumber growth compared with a monoculture of a single strain. Thus, it is of great significance to lay the foundation for the creation of a novel *Trichoderma* biofungicide or biomanure to resist cucumber *Fusarium* wilt and promote cucumber growth.

**Supplementary Information:**

The online version contains supplementary material available at 10.1186/s12934-022-01959-2.

## Background

In nature, microorganisms commonly exist in the form of microbial communities to maintain microecological balance in healthy soil through ecological niche complementarity, functional complementarity, metabolic mutual maintenance, and gene interaction [1–3]; however, it is difficult to replicate the microflora in the natural microbial community completely in the industrial production of biocontrol agents. Traditionally, biocontrol agents are usually prepared with a single strain through monoculture; in recent decades, some multiple strain agents have been used in agriculture but are prepared as simple mixtures [4]. Although an agent with a single strain or a simple mixture of two or more strains can produce a range of biochemical components related to plant disease biocontrol and plant promotion, the agent is universally insufficient to challenge complicated conditions, e.g., multiple plant pathogens present for infection in fields and impacts under diversified environmental stress factors. Therefore, the purposefully combined multiple microorganisms [5, 6], in which consortia contain multiple microbes or strains, which are constructed based on synthetic biology, enable the generation of more complex interactions with the plant than single microbes or strains and thereby achieve synergistic or comprehensive effects in the promotion of plant growth and plant disease prevention in agriculture [7]. As a result, it is particularly important to design a combination of microflora for disease control, even under diversified stress conditions.

*Trichoderma* spp. are widely distributed fungi and commonly used as biocontrol against a range of plant diseases in corn [8], wheat [9], cucumber, etc. [10]. *Trichoderma* has advantages, particularly in the control of crop soil-borne diseases. At present, there are more than 400 *Trichoderma* biological fungicides, and a large number of biological fertilizer products are used globally in agriculture; currently, most biocontrol or biofertilizers containing *Trichoderma* have a single strain or two strains in a simple mixture, and their control effects are not high enough and usually not stable. To overcome this problem, the development of combined multiple strains with complementary effects in pathogen antagonism, environmental stress tolerance, plant promotion, etc., is greatly needed. For example, the product Rootshield of BioWorks in the United States is prepared by the combination of two strains of *Trichoderma* with complementary characteristics. The control effect and growth-promoting effect of combined strain products were better than those of single strain products on plant diseases. However, most of the current multi-strain products are a kind of simple mixture of strains in monoculture, without consideration of complementary relationships among strains in antagonism, stress tolerance, nutrition, metabolism and plant promotion; in particular, co-culture technology is not commonly used on an industrial scale, thus leading to less synergistic action achieved even though multiple strains are applied in an agent.

*Trichoderma* has a remarkable diverse metabolism capable of catabolizing a wide variety of substrates as well as producing a wide variety of secondary metabolites(SMs), of which those most studied are peptaibols, polyketides, pyrones, terpenes and diketopiperazine-like compounds, etc. Many of these compounds are closely involved in biological control and plant growth promotion [11, 12]. Genome sequencing of *Trichoderma* strains in monoculture shows that most of the biosynthetic gene clusters related to secondary metabolites are hidden (transcriptional silencing) or expressed at a very low level under general laboratory conditions, suggesting that these silent metabolic pathways need to be stimulated in some way before they are expressed to produce new metabolites or adequate amounts of metabolites with varied functions. To activate the silent biosynthetic pathway of any strain, the interaction between multiple strains in culture is a prerequisite to induce upregulation of major compound-related genes of all strains involved. Co-culture technology refers to the co-culture of two or more kinds of cells or microorganisms in the same environment, which was first applied to cell co-culture and then gradually applied to microbial co-culture. There are complex ecological relationships among strains in microbial co-culture systems, including synergistic metabolism, competition inhibition, induced activation, gene transfer [13], and quorum sensing [14]. From the application perspective, co-culture involves growing all compatible microbes or strains in the same culture system where the complex interaction between biocontrol microorganisms occurs, aiming to reach the increased yield of active secondary metabolites, including antibiotics and plant growth regulators [15, 16].

Shobha et al. [17] made co-cultures with one *T. harzianum* and two strains of *Trichoderma reesei*, the co-culture products were identified to have a strong binding affinity to the two target proteins of rice bacterial blight, thus inhibiting the activity of the proteins and leading to strong biological control and growth-promoting potential. Iluyemi et al. [18] conducted co-culture with *Aspergillus niger* with *T. harzianum*, *Trichoderma longissimum* and *Trichoderma koningii*, which significantly enhanced the activity of mannan-degrading enzymes and had no significant effect on biomass, indicating that multistrains of *Trichoderma* can form a consortia with compatible interactions between constituent strains.

This study aimed to design and screen consortia composed of multi-*Trichoderma* strains for co-culture on the principle of complementation in function and characteristics related to antagonism, reproductive ability, growth promotion and stress tolerance. To achieve this purpose, a series of assays on *Trichoderma* mycelium growth rate, antagonistic activity, tube cucumber seedling growth and cucumber seed germination rate were used to comprehensively evaluate consortia co-culture and thereby determine the optimal consortia for co-culture. Thus, the study will provide an integrated method to design and screen multi-*Trichoderma* strain consortia for co-culture underlining the preparation of a novel biofungicide or microbial manure against vegetable *Fusarium* wilt.

## Results

### Evaluation of *Trichoderma* strain antagonism

The antagonistic activities of *Trichoderma* were assayed by evaluating the ability of strains that are antagonistic to pathogens, their competition capability, and their chitinase and neutral protease activities. As shown in Table 1, there were differences in the inhibitory effect of 15 strains on antagonistic activities against various pathogens. The inhibitory effects of strain T-10440 against *Botrytis cinerea*, *F. oxysporum*, and *Magnaporthe grisea* were 58.19%, 59.98% and 66.64%, respectively, which were significantly higher than those of the other strains.

**Table 1:** The antagonistic characteristic of *Trichoderma* strains

Strain and code	*B. cinerea *(%)	*M. grisea *(%)	*F. oxysporum *(%)	Competition (cm^2^)	Chitinase (U/mL)	Protease (U/mL)
*T. asperellum *(1009)	52.17 ± 2.22^c^	52.03 ± 1.57^e^	64.71 ± 1.18^def^	0.00 + 0.00^i^	19.52 + 0.15^e^	1.99 + 0.14^ k^
*T. atroviride (3403)*	52.22 ± 0.76^c^	55.81 ± 0.76^bc^	65.36 ± 0.76^cde^	1.22 + 0.14^ef^	22.08 + 0.72^bc^	2.22 + 0.14^jk^
*T. atroviride (10039)*	52.49 ± 1.46^c^	53.2 ± 1.46^de^	62.82 ± 1.46^f^	5.23 + 0.02^b^	20.63 + 0.62^cde^	16.12 + 0.18^c^
*T. atroviride (10043)*	52.71 ± 1.64^c^	53.41 ± 1.64^de^	67.26 ± 1.64^c^	6.35 + 0.06^a^	15.34 + 0.69^f^	5.75 + 0.16^ g^
*T. harzianum (10289)*	43.83 ± 0.8^e^	56.81 ± 0.8^b^	71.99 ± 0.8^a^	2.67 + 0.24^ cd^	22.32 + 2.04^b^	7.77 + 0.15^f^
*T. atroviride (10362)*	43.83 ± 0.77^e^	42.36 ± 0.77^ g^	60.73 ± 0.77^ g^	1.36 + 0.27^f^	24.90 + 1.01^a^	9.19 + 0.23^e^
*T. asperellumn (10420)*	53.41 ± 0.74^bc^	59.89 ± 0.74^a^	66.55 ± 0.74^ cd^	0.74 + 0.03^ h^	20.48 + 0.43^cde^	8.05 + 0.18^f^
*T. asperellum (10440)*	58.19 ± 0.59^a^	59.98 ± 0.59^a^	66.64 ± 0.59^ cd^	1.45 + 0.20^f^	15.41 + 1.72^f^	42.80 + 0.28^a^
*T. asperellum (10539)*	48.97 ± 0.86^d^	54.37 ± 0.86^ cd^	69.62 ± 0.86^b^	1.96 + 0.15^e^	20.89 + 0.51^bcde^	2.81 + 0.28^i^
*T. harzianum (10569)*	57.99 ± 0.67^a^	48.22 ± 0.67^f^	66.44 ± 0.67^ cd^	2.50 + 0.16^d^	25.60 + 1.08^a^	31.88 + 0.2^b^
*T. aureoviride (10644)*	47.82 ± 1.74^d^	30.1 ± 1.74^i^	45.95 ± 1.74^ h^	2.82 + 0.04^c^	24.51 + 0.85^a^	5.78 + 0.18^ g^
*T. harmatum (12048)*	48.74 ± 1.6^d^	36.8 ± 1.6^ h^	63.76 ± 1.6^ef^	1.06 + 0.05^ g^	21.47 + 0.98^bcd^	11.06 + 0.4^d^
*T. viride (12057)*	48.93 ± 1.8^d^	48.55 ± 1.8^f^	65.36 ± 1.8c^de^	1.90 + 0.06^e^	24.05 + 2.31^a^	2.48 + 0.23^j^
*T. asperelloides (Z4-1)*	55.49 ± 3.75^ab^	49.02 ± 0.79^f^	59.41 ± 1.48^ g^	0.55 + 0.08^ h^	20.02 + 0.36^de^	5.01 + 0.18^ h^
*T. asperellum (10264)*	50.16 + 1.01^f^	50.28 + 0.77^ fg^	48.44 + 0.74^ h^	1.35 + 0.16^f^	13.42 + 1.13^ g^	1.54 + 0.20^ l^

Results are average of five replicates for each treatment; the values given are the standard error of the mean. Different superscripts in the same column are significantly different (P < 0.05) based on the ANOVA

In addition, it was seen clearly that there were obvious differences in the competitive effect of 15 *Trichoderma* strains against *F. oxysporum*. Most *Trichoderma* strains can cover colonies of *F. oxysporum,* and strain T-10043 had the strongest competitive effect against *F. oxysporum* at 3 days (Additional file 1: Fig. S1a) when covered pathogen colonies reached an area of 6.35 cm^2^, revealing better antagonistic performance than that of other strains (Table 1).

The activities of some functional enzymes, including chitinase and neutral protease, were compared between 15 *Trichoderma* strains. The activities of chitinase in strains T-0569, T-10362, T-10644 and T-12057 were elevated to 24.51, 24.90, 24.51 and 24.05 U/mL, respectively (Table 1 and Additional file 1: Fig. S1b). T-10440 had the strongest ability to produce neutral protease, and the enzyme activity of protease was 42.80 U/mL (Table 1 and Additional file 1: Fig. S1c).

### Evaluation of growth, reproduction and stress tolerance of *Trichoderma*

**Fig. 1:**
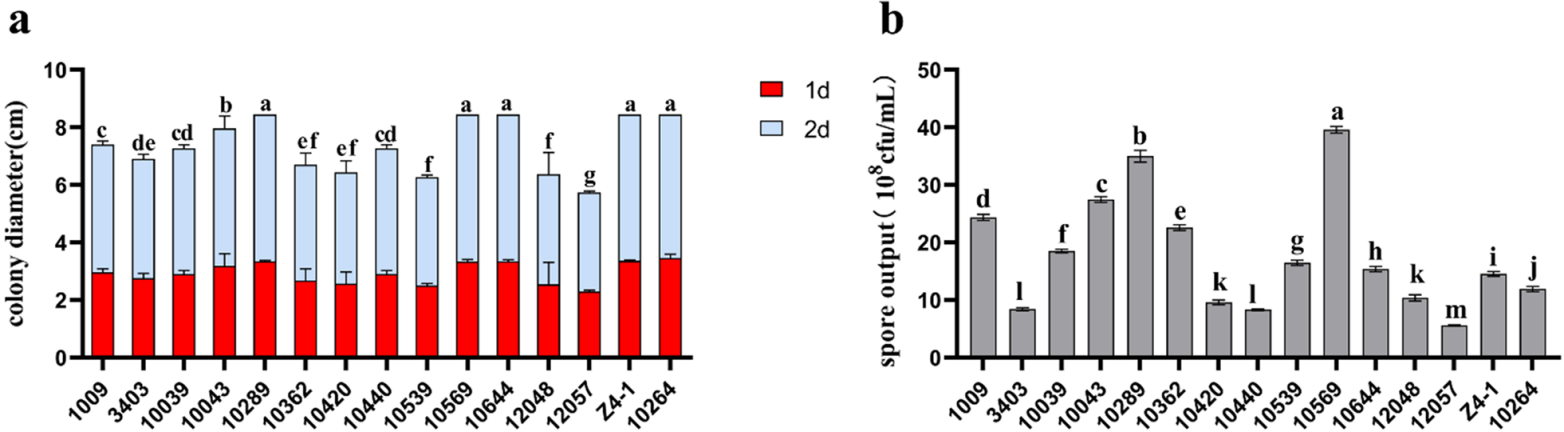
Evaluation of growth and reproduction of *Trichoderma* strains. **a** The growth rate of the *Trichoderma* strain on PDA plates for 1 day and 2 days. **b** Sporulation amount of *Trichoderma* strains on PDA plates. Data are the mean of 5 replicates for each treatment; the value is the standard error of the mean. Different letters above the bars indicate significant differences (P < 0.05) based on ANOVA

The mycelium growth, sporulation and stress tolerance of 15 strains of *Trichoderma* were evaluated in a plate assay. Figure 1a shows that all *Trichoderma* strains were able to grow over the whole PDA plate (90 cm in diameter) within 5 days. The growth rate of strains T-10264, T-10289, T-10569, T-10644 and T-Z4-1 was grown over the whole plate in 3 days. Figure 1b reveals obvious differences in the sporulation rate between 15 *Trichoderma* strains grown on PDA plates. Except for strains T-10289 and T-10644, all other strains were slower in sporulation on PDA.

**Fig. 2:**
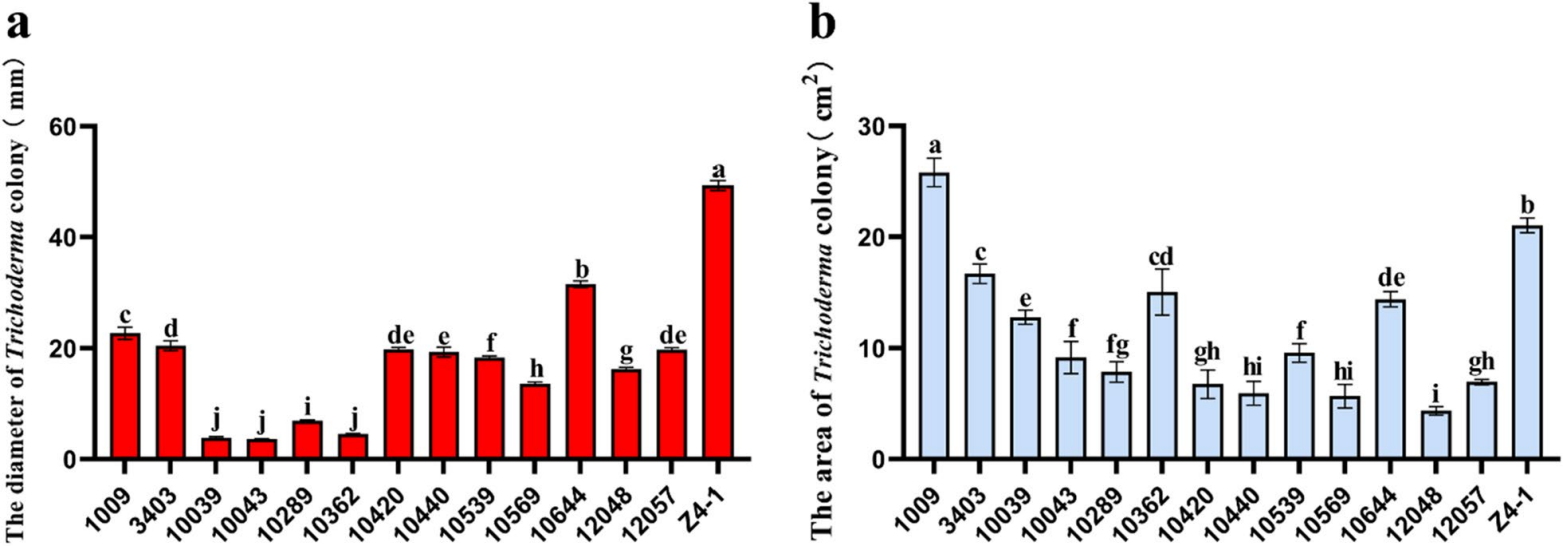
Detection of stress tolerance of *Trichoderma* strains. **a** Colony growth of *Trichoderma* strains on PDA medium supplemented with 1 M NaCl. **b** Colony growth of different *Trichoderma* strains on PDA medium at 36 °C. The results are the means of 5 replicates for each treatment; the value is the standard error of the mean. Different letters above the bars indicate significant differences (P < 0.05) based on ANOVA

According to the high temperature and salt tolerance evaluation threshold and standard available for *Trichoderma* proposed by Wang Qiangqiang [19], only strains T-Z4-1 and T-10264 showed medium temperature tolerance (Fig. 2a) [19]. Only T-1009 and T-10264 showed high salt tolerance (Fig. 2b), and the other strains exhibited low tolerance to the stress condition.

### Evaluation of the ability of *Trichoderma* strains to promote cucumber seedling growth

**Fig. 3:**
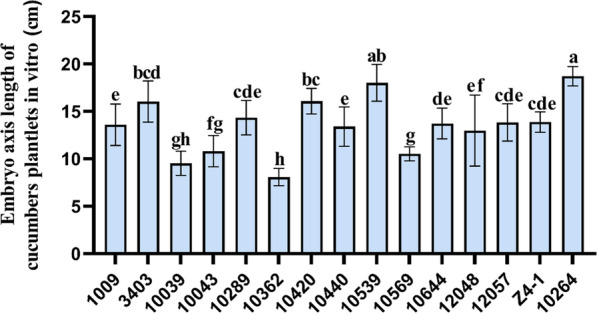
Effects of *Trichoderma* strains on the growth of cucumber plantlets. The results are the means of 5 replicates for each treatment; the value is the standard error of the mean. Different letters above the bars indicate significant differences (P < 0.05) based on ANOVA

Among the strains assayed, strain T-10264 had the strongest promotion of the growth of cucumber test tube plantlets, and the hypocotyl length of cucumber plantlets was 18.715 ± 1.17 cm (Fig. 3). Next was strain 10539 at 18.016 ± 1.94 cm (Fig. 3).

### Design of *Trichoderma* multi-strain consortia for co-culture and evaluation

**Fig. 4:**
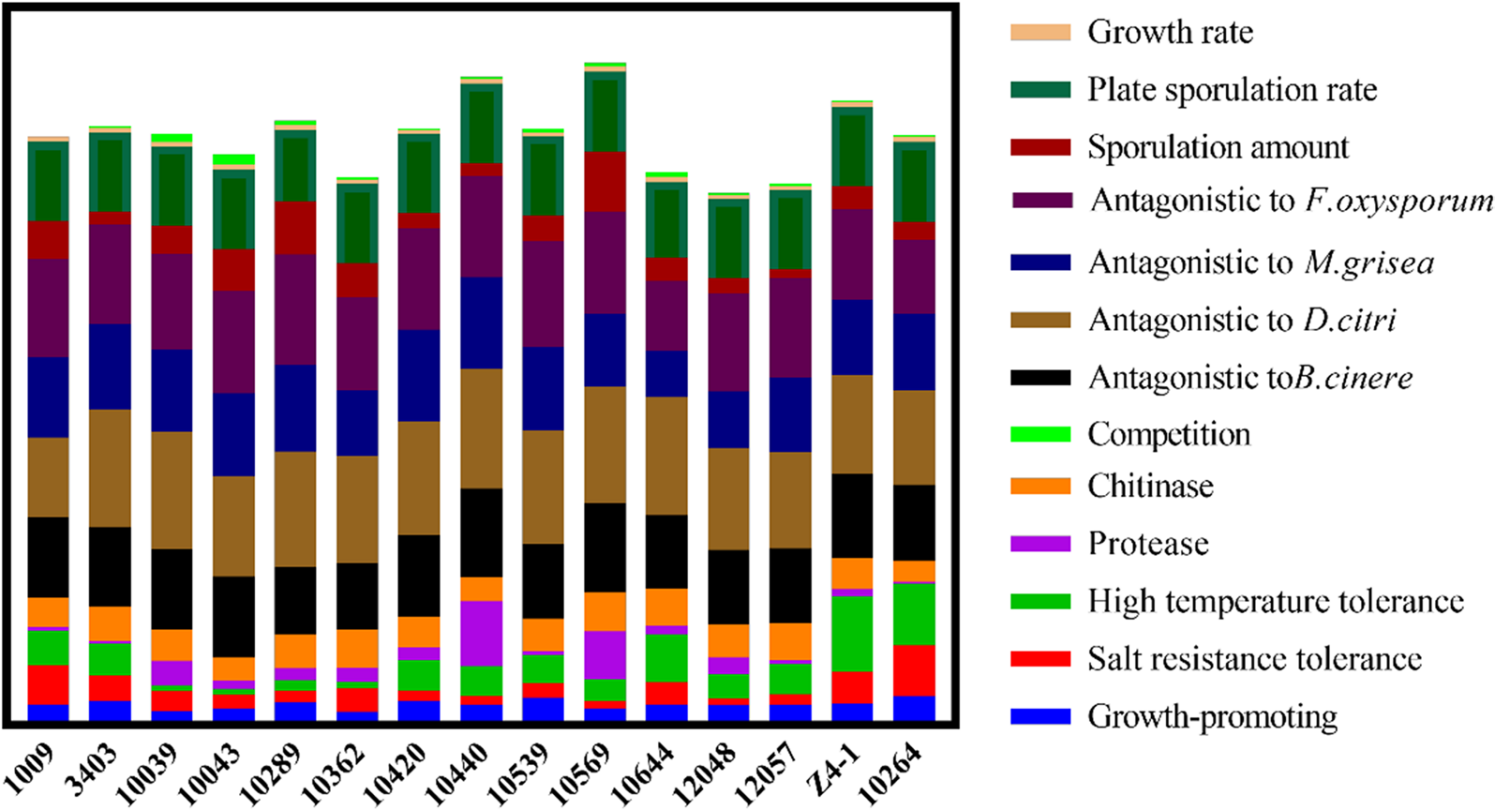
Comprehensive evaluation of the growth, reproduction, tolerance and antagonism of different *Trichoderma* strains

Based on 14 evaluation indicators, including antagonistic activity, stress tolerance and plant growth-promoting effects (Fig. 4), T-Z4-1 featured medium tolerance to high-temperature stress, T-1009 had high tolerance to high-salt stress, T-10043 had high competitivity to the pathogen, T-10440 had high antagonistic activity, T-10569 had high chitinase activity, and T-10264 had high plant growth-promoting effects and were all selected to design varied strain combinations, numbered T1, T2, T3, T4, T5, and T6, respectively.

Six *Trichoderma* strains were combined by 2 strains, 3 strains, 4 strains, 5 strains and 6 strains. The filtrate of the co-culture combinations was evaluated in three aspects: the dry weight of co-cultured *Trichoderma* mycelium, the inhibition rate against *F. oxysporum* and the plant growth-promoting effect.

**Fig. 5:**
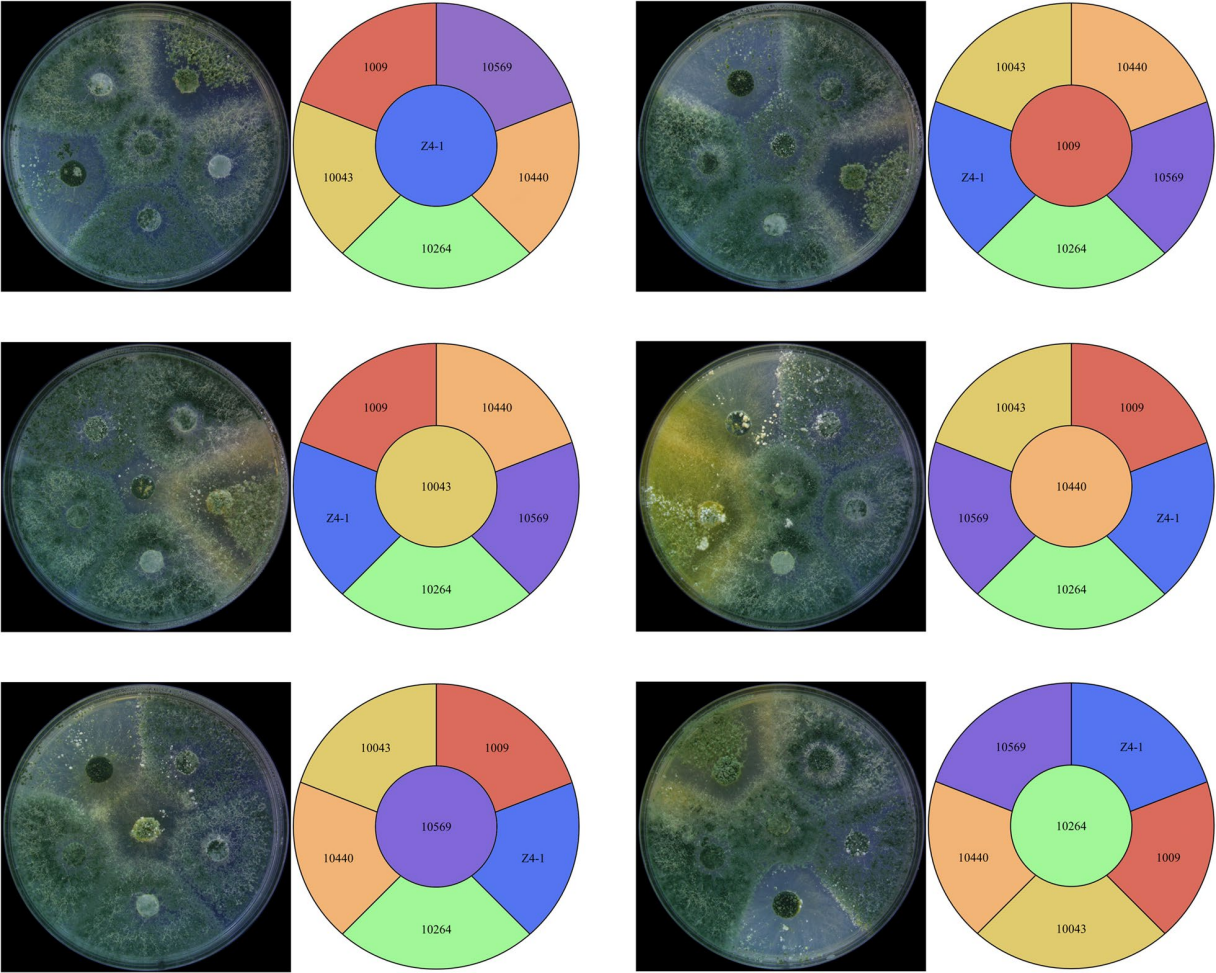
Detection of compatibility among six *Trichoderma* strains

To achieve a balanced co-culture between selected *Trichoderma* strains, it was necessary to determine the compatibility between these strains before co-culture was performed. These strains were cultured on a PDA plate for 5 days according to the above combination, and the results showed that, except for the obvious incompatibility among T-10043 and other strains, the other strains showed compatibility (Fig. 5).These results provide a good foundation for subsequent co-cultivation.

**Fig. 6:**
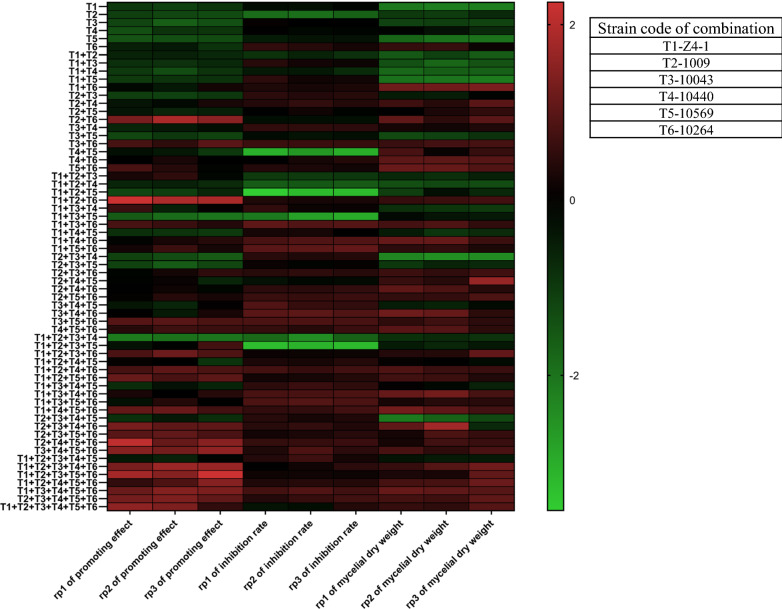
Functional evaluation of consortia of six *Trichoderma* strains. rp1: repeat 1; rp2: repeat 2; rp3: repeat 3. The results are the means of 3 replicates for each treatment; the value is the standard error of the mean. Different letters above the bars indicate significant differences (P < 0.05) based on ANOVA

The optimal combination in 57 Trichoderma (Fig. 6) co-culture combinations was selected according to their comprehensive performance in the dry weight of *Trichoderma* mycelium, co-culture filtrate inhibition rate to *F. oxysporum* and plant growth-promoting effect, and monocultures of a single strain were set up as controls. Compared with a monoculture of a single strain, the co-culture of consortia was significantly changed in the three aspects mentioned above; interestingly, it was found that more strains in consortia did not necessarily lead to improved performance. Comparatively, the complementarity among strains was viewed as the most important trait for successful co-culture, particularly in biocontrol and plant promotion as well as related genes and metabolism pathways.

### Evaluation of *Trichoderma* culture filtrate function

**Fig. 7:**
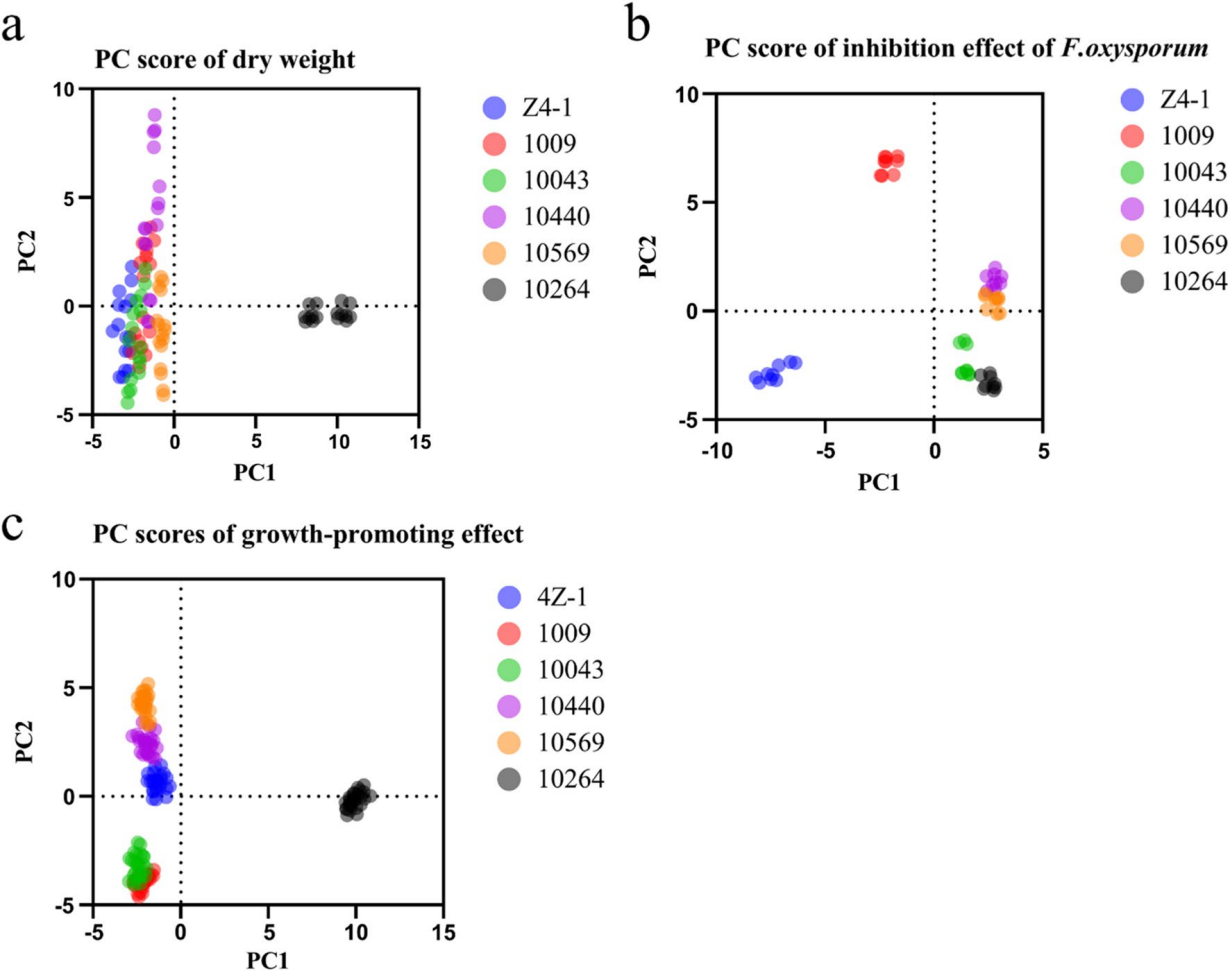
PCA of *Trichoderma* consortia co-culture. **a** Effect of *Trichoderma* strain consortium co-culture filtrate on the dry weight of mycelium. **b** *Trichoderma* strain consortium co-culture filtrate inhibitory effect on *F. oxysporum*. **c** Effect of *Trichoderma* strain consortium coculture filtrate on the plant growth-promoting effect of cucumber. Data are the means of 3 replicates for each treatment; the value is the standard error of the mean. Different letters above the bars indicate significant differences (P < 0.05) based on ANOVA

*Trichoderma* mycelial growth: The mycelial dry weight of consortia T1 + T6 (T-Z4-1 + T-10264) was 9.19 ± 0.95 mg/mL, which was the highest among all combinations, and the mycelial dry weight of consortia T1 + T2 + T3 + T4 + T5 + T6 (T-Z4-1 + T-1009 + T-10043 + T-10440 + T-10569 + T-10264) was 8.23 ± 0.15 mg/mL, which was lower than that of consortia T1 + T6 (Fig. 6). The results showed that increasing the number of strains did not necessarily increase the dry weight of mycelia. PCA was performed on the effect of the increase in the number of strains in the consortium co-culture filtrate on the mycelial dry weight. The principal component analysis function formula y_a_ = 0.0916y_1_ + 0.2754y_2_ + 0.2043y_3_ + 0.2664y_4_ + 0.1336y_5_ + 0.1508y_6_ was obtained. T2 (strain 1009) had the highest contribution to mycelial dry weight in the combination (Fig. 7a).

Antagonistic effect on pathogen: It was revealed that the inhibition rate to *F. oxysporum* with consortia co-culture filtrate of T1 + T5 + T6T-(Z4-1 + T-10569 + T-10264) was 96.33 ± 0.26%, the highest of the 57 combinations. However, the inhibition rate of consortia co-culture filtrate of T1 + T2 + T3 + T4 + T5 + T6 to *F. oxysporum* was 93.17 ± 1.15%, which was lower than that of consortia co-culture T1 + T5 + T6 (Fig. 6), indicating that simply increasing the number of strains in consortia does not necessarily increase the rate of inhibition to *F. oxysporum*. Furthermore, the influence degree of each strain in the consortia co-culture filtrate calculated by PCA was obtained using the formula y_b_ = 0.1595y_1_ + 0.1458y_2_ + 0.2223y_3_ + 0.0631y_4_ + 0.0474y_5_ + 0.2385y_6_. T6(T-10264) had the highest contribution to the inhibition rate of *F. oxysporum* in the combination (Fig. 7b).

Promotion of cucumber plantlet growth: The fresh weight of cucumber test tube plantlets treated with consortia co-culture filtrate of T1 + T2 + T6(Z4-1 + 1009 + 10264) was 0.45 ± 0.03 g, which was the highest among the 57 combinations. The fresh weight of cucumber test tube plantlets treated with consortia co-culture filtrate of T1 + T2 + T3 + T4 + T5 + T6 was 0.36 ± 0.06 g, lower than that of the co-culture filtrate of T1 + T2 + T6 combinations (Fig. 6), which demonstrated that increasing the number of strains did not necessarily increase the cucumber growth-promoting effect. The contribution of each strain in the co-culture system was calculated by PCA using the formula y_c_ = 0.0703y_1_ + 0.1973_y2_ + 0.147y_3_ + 0.1815y_4_ + 0.1367y_5_ − 0.1148y_6._ T2 had the highest contribution to the synergistic effect in the consortia for the promotion of cucumber test tube plantlet growth (Fig. 7c).

**Fig. 8:**
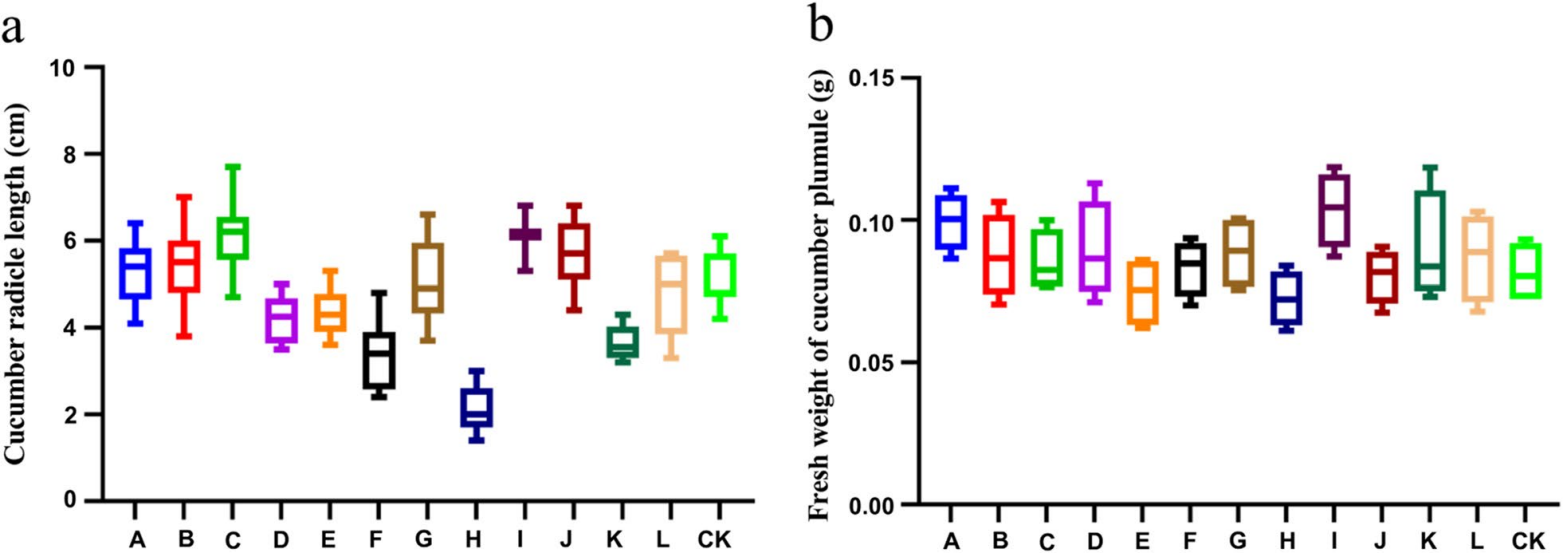
Determination of the optimal Trichoderma consortia by cucumber seedling growth-promoting assay. **a** Effects of *Trichoderma* consortia co-culture filtrate on cucumber radicle length**; b** Fresh weight of cucumber plumule. **A** T1 + T2 + T5 + T6 original filtrate; **B** T1 + T2 + T5 + T6 original filtrate diluted 50 times; **C** T1 + T2 + T5 + T6 original filtrate diluted 100 times; **D** T1 + T2 + T5 + T6 original filtrate diluted 200 times; **E** T2 + T3 + T4 + T5 + T6 original filtrate; **F** T2 + T3 + T4 + T5 + T6 original filtrate diluted 50 times; **G** T2 + T3 + T4 + T5 + T6 original filtrate diluted 100 times; **H** T2 + T3 + T4 + T5 + T6 original filtrate diluted 200 times; **I** T1 + T2 + T6 original filtrate; **J** T1 + T2 + T6 original filtrate diluted 50 times; **K** T1 + T2 + T6 original filtrate diluted 100 times; **L** T1 + T2 + T6 original filtrate diluted 200 times; CK: PD medium. Data are the means of 5 replicates for each treatment; the value is the standard error of the mean. Different letters above the bars indicate significant differences (P < 0.05) based on ANOVA.

To determine the optimal consortia co-culture filtrate, the consortia that ranked first in the growth promotion of cucumber test tube plantlets was selected for further evaluation of the co-culture filtrate effect on the cucumber seed germination assay. Among consortia T1 + T2 + T6, T2 + T3 + T4 + T5 + T6(T-1009 + T-10043 + T-10440 + T-10569 + T-10264) and T1 + T2 + T5 + T6(T-Z4-1 + T-1009 + T-10569 + T-10264), there were no significant differences in the effect of the original filtrate on cucumber seed germination. However, the co-culture filtrate from consortia T1 + T2 + T5 + T6 diluted 100 × was significantly better than those of other consortia co-culture filtrates in the enhancement of the length of the radicle; thus, consortia T1 + T2 + T5 + T6 was selected as the best one for the follow-up experiment (Fig. 8).

### Analysis of components in *Trichoderma* culture filtrate

**Fig. 9:**
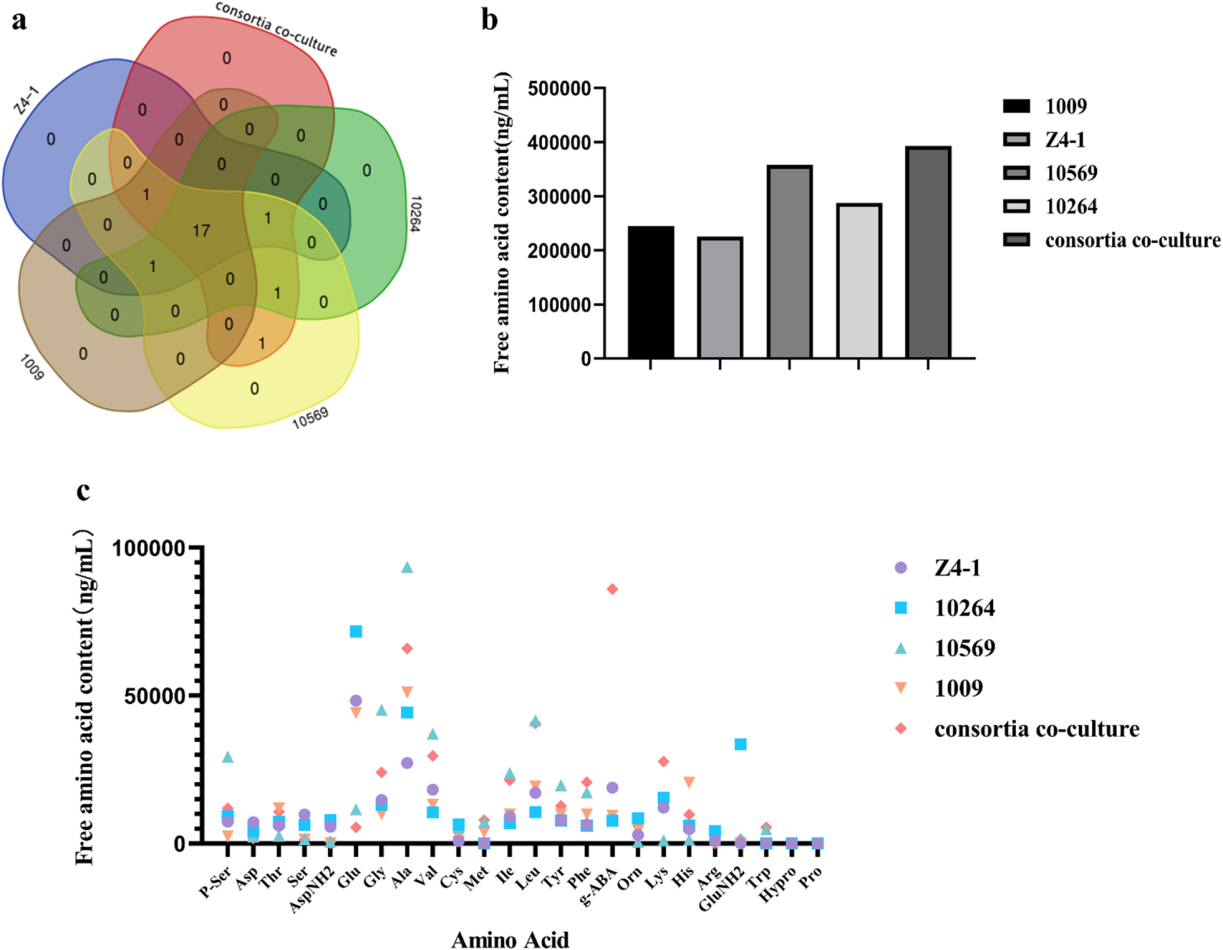
Effects of co-culture and monoculture on the yield and species of amino acids of *Trichoderma* strains. **a**Yield and kinds of free amino acids in *Trichoderma* co-culture filtrate. **b** Yield and kinds of free amino acids in *Trichoderma* co-culture filtrate. Data are the means of 3 replicates for each treatment; the value is the standard error of the mean. Different letters above the bars indicate significant differences (P < 0.05) based on ANOVA

The content of free amino acids in *Trichoderma* co-culture filtrate was significantly higher than those from a single *Trichoderma* strain in monoculture (Fig. 9a). The yield of free amino acids in the filtrate from consortia T1 + T2 + T5 + T6 was 392,841.4 ng/mL, which was 60.55%, 74.38%, 9.83% and 36.68% higher than that of T-Z4-1, T-1009, T-10569 and T-10264, respectively, indicating that consortia T1 + T2 + T5 + T6 was the best one in the production of amino acids in co-culture.

The differential amino acid in the content between co-culture filtrates from different consortia was γ-aminobutyric acid (g-ABA). However, the species of free amino acids produced in co-culture were not much different, and unique ones were not found, although the co-culture significantly improved the production of amino acids.

### Co-culture filtrate of optimal *Trichoderma *consortia to improve cucumber seedling growth

**Fig. 10:**
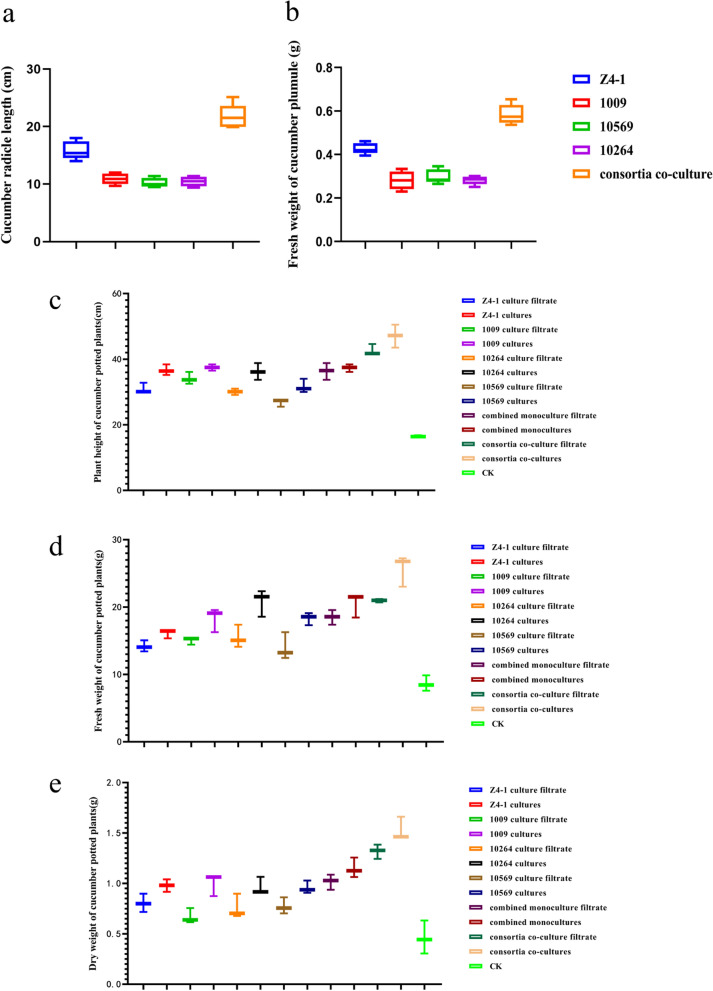
Effects of optimal consortia co-culture filtrates on cucumber seedling growth. **a** *Trichoderma* consortia co-culture filtrate on cucumber plantlet radical length. **b** *Trichoderma* consortia co-culture filtrate on cucumber plantlet plumule fresh weight. **c **Effects of *Trichoderma* consortia co-culture filtrate on the plant height of cucumber seedlings in pot cultivation. **d** *Trichoderma* consortia co-culture filtrate on fresh weight of cucumber seedlings in pot cultivation. **e** *Trichoderma* consortia co-culture filtrate on dry weight of cucumber seedlings in pot cultivation. Data are the means of 3 replicates for each treatment; the value is the standard error of the mean. Different letters above the bars indicate significant differences (P < 0.05) based on ANOVA

The cucumber growth-promoting effects presented by the co-culture filtrate of the optimal consortia relative to the single strain culture filtrate were observed based on the growth changes of cucumber radicles and plumules. It was revealed that the filtrates from the co-culture of consortia generated a better promotion to test tube cucumber plantlets than that of the single strain culture filtrate. The length and fresh weight of the test tube plantlets in the treatment of the T1 + T2 + T5 + T6 consortium co-culture filtrate were 21.72 ± 2.11 cm (Fig. 10a) and 0.584 ± 0.046 g (Fig. 10b), respectively, which were significantly higher than those of the single strain culture filtrate.

Results suggested that co-culture filtrate of optimal consortia took significant advantage over the monoculture in cucumber seedling promotion effect and even better than that of the monoculture filtrate from simply combined monocultures, in the treatment with co-culture filtrate of consortia from T1 + T2 + T5 + T6, cucumber seedling plant height reached 47.07 ± 2.50 cm, fresh weight of pant above-ground was 25.68 ± 2.31 g, and dry weight of pant above-ground was 1.53 ± 0.12 g, which were significantly higher than in the treatment with single strain monoculture or simply combined monocultures. Regardless of monoculture or co-culture, the cultures containing mycelia and spores performed better in plant promotion effect than culture filtrate without mycelia and spores, implying that some synergistic action of *Trichoderma* mycelium and its metabolites occurred in the promotion of plant growth. Thus, it was confirmed that metabolites from the *Trichoderma* four strains co-culture were superior to those of a single strain in terms of promotion of cucumber seedling growth.

### Field experiments of cucumber growth-promoting effects

**Fig. 11:**
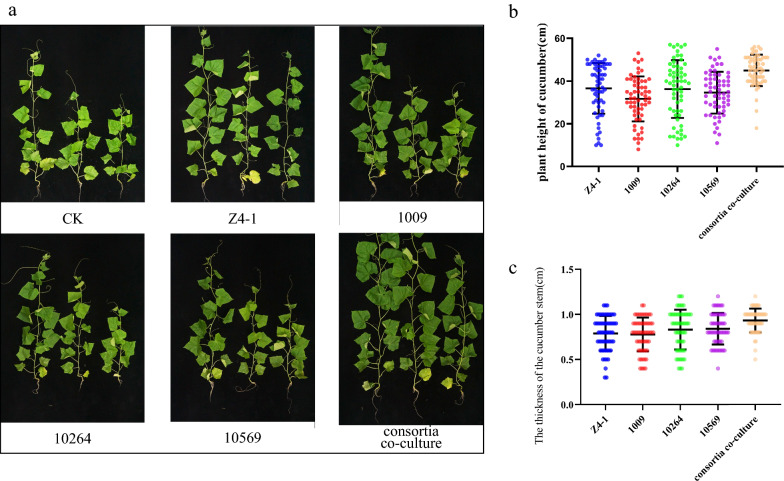
The effect of co-culture of consortia on the growth of cucumbers in the field. **a** Cucumber growth chart in the field. **b** The plant height of cucumber 20 days after transplanting. **c** The diameter stems of cucumber 20 days after transplanting. The results are the means of 60 replicates for each treatment; the value is the standard error of the mean. Different letters above the bars indicate significant differences (P < 0.05) based on ANOVA

The results of field experiments showed that the growth promotion effect of *Trichoderma* co-culture fermentation filtrate on cucumber test tube seedlings was significantly better than that of the single culture fermentation filtrate. Twenty days after transplanting, the average plant height of cucumbers treated with culture fermentation filtrate was 45.03 cm (Fig. 11a and b), and the average stem diameter was 8.26 cm (Fig. 11a and c), which was higher than that of cucumbers treated with single culture fermentation filtrate. Compared with T-Z4-1, T-1009, T-10264 and T-10569, the plant height of cucumber was increased by 22.99%, 42.06%, 24.18% and 30.09%, respectively, and the stem diameter was increased by 16.59%, 18.83%, 13.65% and 14.70%, respectively.

## Discussion

In this study, a group of *Trichoderma* strains with excellent characteristics in antagonistic activities, mycelial growth and sporulation, stress tolerance and plant growth-promoting ability were screened through comprehensive evaluation at different levels. These strains have also been identified as nontoxic through animal tests (Additional file 6), which thus could be used as a source of nontoxic metabolites with plant promotion and biocontrol functions. Based on these excellent strains, a design technique was first applied to establish multiple consortia of *Trichoderma* strains with complementary and synergistic effects on biological characteristics, biocontrol and plant promotion functions. The cyclic iterative upgrade model of Design-Test-Learn of microbial assembly design is referred to in the combination design of microbes. The method presented in our study allows researchers to add or replace any specific strains to the *Trichoderma* consortia, leading to an upgrade in the microbial components of each consortium [20]. In addition, a fast evaluation method, cucumber test tube plantlets, was set up for *Trichoderma* strains and consortia evaluation in the potential of plant growth promotion. It usually requires 5 days to complete the evaluation process. Jiang Xiliang et al. [21] developed a method to evaluate the biocontrol effect of *Trichoderma* by test tube plantlets. Wang Qiangqiang et al. [22] developed a similar method to evaluate mycelial growth, biocontrol effects, and stress resistance.

At present, the majority of *Trichoderma* agents in the world are composed of a single strain or double strains, which are usually unstable in biocontrol and plant promotion performance in natural conditions [23]. To overcome this problem, we established 4 strains-consortia, which performed excellently in biocontrol and plant promotion-related characteristics since the individual constituent strains were sourced from different ecological systems involving crop farming fields, forests, wetlands and orchards; therefore, the consortia of four strains were made a feature with wide-spectrum adaptability to environmental stress, which would make the consortia more stable during its application in agriculture. In addition, the complementary and synergistic effects formed in the four strains could further enhance the versatility of the consortia.

Co-culture techniques are an alternative approach to explore novel metabolites or increase some targeted metabolites through interactions between different species of microbes in the same culture system [24]; for example, the co-culture of *T. asperellum* and *Bacillus amylolifasicens* generated higher amounts of amino acids and lipids compared with a single strain in monoculture. Similarly, our study also demonstrated that the co-culture filtrate from 4-*Trichoderma* strains-consortia showed a higher synergistic effect than that from a single strain monoculture in cucumber seed germination and seedling growth and was even better than that of mixed filtrates from the separate culture of single strains.

Compared with a single strain in monoculture, co-culture of multiple strains is better than single strain culture, hinting that some silent genes or metabolic pathways might be activated in the process of the co-culture process. *Trichoderma-Bacillus* strain co-cultures may produce antibiotic secondary metabolites or growth-promoting substances that are never found in a single strain culture. In this study, the yield of γ-aminobutyric acid (GABA) [25–27] in co-cultures of *Trichoderma* multistrain consortia was higher than that in single strain cultures. GABA is related to a variety of stress and defence responses in plants [26]. GABA increases with the stimulation of plants, which is an effective mechanism for plants to respond to a variety of external changes and internal stimuli. Exogenous spraying of GABA can improve the resistance of plants to environmental stresses such as low temperature, waterlogging, salinity, and drought [28]. Qiang et al. [29] co-culture of *Bacillus* and *Trichoderma* also induced the production of a large number of amino acids and other organic acids. Karuppiach et al. [30] established a *Trichoderma-Bacillus* co-culture [31–33] system through response surface design, in which *Trichoderma* induced the production of *Bacillus* macrolide and macrolide antibiotics and specifically produced eurycomanone (with medical value). At the same time, it induced the upregulated expression of *Bacillus* chitinase, glucanase, cellulase, and protease genes [34] and produced IBA plant growth regulators. Comparatively, *Bacillus* can induce the more diverse biocontrol-related genes expression in *Trichoderma* (Qiang et al. [29]).

Chen Kai et al. Co-cultured *T. atroviride* HB20111, *T. harzianum* LTR-2 and TW21990 [35] in liquid, and the co-fermentation filtrate had the highest inhibition rate of 71.83% on *F. graminearum* spore germination, which was 6.13% and 19.53% higher than that of the single strains HB20111 and TW21990, respectively [36]. The seed soaking with 500× dilution of co-culture fermentation filtrate achieved the control of cucumber *Fusarium* wilt and increased cucumber seedlings’ fresh weight by 73.65% and 35.64%, respectively, which were 6.76%, 9.46% and 11.54%, 13.31% higher than those of single strain HB20111 and TW21990, respectively.

In the field experiments, it was generally confirmed that the co-culture metabolites from consortia consisting of T-Z4-1, T-1009, T-10264 and T-10569 were able to promote cucumber growth investigated at 20 days after transplanting. It was supposed that the increased plant growth-regulated metabolites in co-culture might contribute to plant promotion; those metabolites usually include ee amino acids, 6-pentyl-2H-pyran-2-one, koninginins, indolebutyric acid (IBA) and indole-3-acetic acid (IAA) [37]. Although the growth promotion effect in the field was relatively improved, due to the small scale of fermentation, there were slight differences between fermentation filtrate. In future research, the co-fermentation scheme will be further optimized to improve product stability.

However, the number of strains in consortia of *Trichoderma* strains is not absolutely and necessarily correlated with the improved function. For example, T1 + T2 + T3 + T4 + T5 + T6 of the strain combinations covered the 6 strains selected in the study, but the final optimal combination was not this combination; instead, T1 + T2 + T5 + T6 was the best combination. Therefore, the combinatorial design of *Trichoderma* is not simply dependent on the increased numbers of strains, more importantly, which relies on symbolic and synergistic interactions of strains through compatible co-metabolism and gene circuit regulation. Oscar et al. [38] co-cultures of two strains of *T. harzianum* led to an inhibitory effect on the development of lateral roots of quinoa. Therefore, in the process of *Trichoderma* co-culture combination design, the effect of co-fermentation must be evaluated from many aspects to avoid negative effects.

## Conclusions

A design method to quickly screen a *Trichoderma* multiple strain consortium was established, and an optimal *Trichoderma* consortium of T-Z4-1, T-1009, T-10569 and T-10264 environmental nontoxicants was featured as the optimal consortium in biocontrol-related functions and plant growth-promoting potential relative to a single strain or strain mixture. The co-culture filtrate contained a higher yield of amino acids than that of single strains in monoculture. Interestingly, γ-aminobutyric acid (GABA) was especially high in the consortia co-culture filtrate, which is known as a crucial substance needed for cucumber seedling growth improvement. However, the quality of consortia of *Trichoderma* strains is only dependent on the number of strains involved, and the compatible and complementary interaction between strains is a priority to establish optimal consortia for co-culture. Taken together, the results, on the one hand, not only offer a tool to design and screen *Trichdoema* strain consortia for co-culture but also allow us to develop a novel biocontrol agent or biofertilizer in the future.

## Materials and methods

### Fungal strains and culture

*Trichoderma* spp., separated from the leaves, peel and bark (121.843239° E, 31.463703° N) of plants in the ecological orange orchard in Yingdong Village, Chenjia Town, Chongming Island, Shanghai, were stored in a laboratory refrigerator at − 80 °C. *Trichoderma* spp. (Additional file 1: Table S1) and *F. oxysporum*, *B. cinerea*, and *Diaporthe citri* were provided by Shanghai Jiao Tong University Culture Preservation Center. *M. grisea* was donated by Professor Chen Gongyou, School of Agriculture and Biology, Shanghai Jiao Tong University.

The spore suspensions of *Trichoderma, F. oxysporum, B. cinerea, D. citri* and *M. grisea* stored in 25% glycerin were inoculated with 5 μL in the centre of the PDA plate at 28 °C and incubated in a thermostatic incubator for 5 days for activation. After activation, those strains were then transferred onto the plate to recultivate them 2–3 times for rejuvenation until the colony morphology was stable (Additional files 2, 3, 4, 5) [34, 39].

### Plant culture conditions

The cucumber seeds used in this experiment were Shenqing No. 1, purchased from Shanghai Funong Seed Industry Co., Ltd.

Cucumber seed germination: Cucumber seeds were disinfected with 75% alcohol for 30 s, washed with sterile distilled water three times, soaked with 3% sodium hypochlorite for 8–10 min, and finally washed with sterile distilled water 3–4 times. The sterilized seeds were evenly placed in sterilized double layer filter paper, the filter paper was placed in a sterilized petri dish, sterile distilled water was added to soak the filter paper, and germination was at 26 °C away from light for 3 days [40].

Cucumber pot cultivation: Cucumber seeds were grown for 3 days per pot. Water was added after sowing until there was water overflowing from the drainage hole at the bottom of the disc. In artificial climate indoor cultivation, the seedling light time was 16 h light/8 h dark, the indoor temperature was controlled from 24 to 25 °C, and 1000 mL of sterile water was added every 3 days. After 3 days, only one seedling per pot was retained for growth. Five replicates were performed per treatment.

### Inoculum preparation

PD liquid culture: A spore suspension was prepared by adding 5 mL sterile distilled water to a 5 days-incubated *Trichoderma* plate, scraping off the *Trichoderma* spores on the plates, and placing them into a 10 mL sterile centrifuge tube (1 × 10^8^ CFU/mL). The 0.5 mL *Trichoderma* spore suspension was transferred into a 250 mL flask containing 100 mL of PD medium incubated in a shaker for 5 days at 28 °C and 200 r/min.

Fermentation medium: Corn flour 50 g/L, potassium dihydrogen phosphate 3.82 g/L, sodium nitrate 1.42 g/L, ammonium sulfate 1.1 g/L, sodium chloride 1 g/L, magnesium sulfate heptahydrate 0.5 g/L, ferrous sulfate heptahydrate 0.0075 g/L, manganese sulfate 0.0025 g/L, zinc sulfate 0.002 g/L, and natural pH.

### Determination of *Trichoderma* growth and reproductive ability

The *Trichoderma* strain disc was taken from the PDA colony edge with a sterilized cork borer (diameter 5 mm) and transferred to the new medium to spread. PDA culture was incubated in the dark at 28 °C for 5 days, during which the colony diameter and spore-producing area were observed and recorded every 24 h. All the spores on the surface of the medium were washed with a certain amount of sterile water at 5 days, and the number of *Trichoderma* spores was counted with a haemocytometer. Each treatment was replicated three times.

### Plate confrontation assay of *Trichoderma* and the pathogen

The discs from the edges of *Trichoderma* and pathogen colonies were taken with a sterilized cork borer (5 mm in diameter) and in turn were placed on PDA Plates 2.5 cm apart. The pathogen was singly grown in PDA as a control, and all inoculated plates were placed in the incubator at 28 °C for 5 days. Then, the inhibition of pathogen growth was determined by measuring the inhibited pathogen colony radius by *Trichoderma*. Each confrontation assay was set to three 3 replicates. The inhibition rate was calculated as follows:

$$ {\text{Inhibition rate}}\left( \% \right) = {{\left( \begin{gathered}  {\text{colony radius of control group}} \hfill \\  - {\text{colony}}\,{\text{radius of treatment group pointing}}\,{\text{centre of the plate}} \hfill \\ \end{gathered} \right)} \mathord{\left/ {\vphantom {{\left( \begin{gathered}  {\text{colony radius of control group}} \hfill \\  - {\text{colony}}\,{\text{radius of treatment group pointing}}\,{\text{centre of the plate}} \hfill \\ \end{gathered} \right)} {{\text{colony radius of control group}} \times 100.}}} \right. \kern-\nulldelimiterspace} {{\text{colony radius of control group}} \times 100.}} $$

### Determination of antagonistic ability of *Trichoderma*

Competitive effect assay: The discs were taken from the edges of activated *Trichoderma* and pathogen colonies with a sterilized cork borer (diameter 5 mm) and inoculated in the same straight Line 2.5 cm apart on PDA plates incubated for 5 days at 28 °C. Trichoderma coverage of the area of pathogen colonies was determined every day, with individual strain-grown plates as a control. Each treatment included 3 plates.

Chitinase activity assay: The chitinase activity of the ferment filtrate was estimated using a chitinase extraction kit (Solarbio, catalogue number BC0820) according to the manufacturer’s protocol. Chitinase active unit definition: The amount of enzyme needed for 1 μmol N-acetylglucosamine production from decomposed chitin by 1 mL culture medium at 37 °C per hour is one unit of enzyme activity (U).

Neutral protease activity assay: The neutral protease activity of the ferment filtrate was estimated using a neutral protease (NP) extraction kit (Solarbio, catalogue number BC2290) according to the manufacturer’s protocol. NP activity unit definition: At 30 °C, 1 mL sample per minute catalytic hydrolysis to produce 1 μmol tyrosine as an enzyme living unit (U).

### *Trichoderma* tolerance to adversity evaluation

Salt tolerance: 1 mol/L NaCl was set as the salt tolerance threshold concentration, and the evaluation grading criteria were made based on *Trichoderma* growth at the threshold concentration.

Low salt tolerance: The colony diameter was less than 50% (< 12.56 cm^2^) of the control colony area after 4 days of incubation at 28 °C; Medium salt tolerance: The colony diameter was 50–70% (12.56–23.75 cm^2^) of the control colony area after 4 days of incubation at 28 °C; High salt tolerance: colony diameter greater than 70% (> 23.75 cm^2^) after 4 days of incubation at 28 °C.

Stress temperature tolerance: A temperature of 36 °C was determined as the threshold temperature for determining the criteria of *Trichoderma t*olerance to stress temperature.

Low-temperature tolerance: The colony diameter was 50% or less of the control after 4 days of incubation at 36 °C (< 4 cm). Medium high-temperature tolerance: The colony diameter was 50–70% (4–5.5 cm) of the control diameter after 4 days of incubation at 36 °C. High-temperature tolerance: The colony diameter was greater than 70% (> 5.5 cm) of the control diameter after 4 days of incubation at 36 °C.

### Evaluation of *Trichoderma* growth-promoting function

*Trichoderma* fermentation culture was centrifuged at 8000 rpm for 10 min to remove mycelia and spores. Then, 0.5 mL of supernatant was mixed with 22 mL of Hoagland’s nutrient solution. Half a sheet of 7 cm filter paper rolled into a barrel was placed in a 5 mL centrifuge tube and set aside after sterilization. Four millilitres of the mixture was placed into the centrifuge tube. Cucumber radicles with shoots of similar size at 3 days were placed into centrifuge tubes and incubated in the dark at 26 °C for 5 days. Five replicates were set per treatment.

### Design for consortia with excellent *Trichoderma* strains for co-culture

#### Evaluation and screening of excellent *Trichoderma* strains

Fifteen functional evaluation indicators: *Trichoderma* plate growth rate, sporulation rate, growth-promoting effect on cucumber test tube plantlets, chitinase production, glucanase production, neutral protease production, nutritional competition, inhibition to *F. oxysporum, M. grisea*, *D. citri* and *B. cinerea*, high-temperature tolerance and salt tolerance.

#### Screening of multiple strain consortia

The excellent strains of *Trichoderma* were selected for the combination of 2, 3, 4, 5 and 6 strains. First, the compatibility was verified by co-inoculation on PDA plates, and then liquid co-culture was carried out. The biocontrol function was characterized by the inhibition rate of *F. oxysporum*, and the growth-promoting function was evaluated using cucumber test tube plantlets and the production feasibility of co-cultured mycelial dry weight, the data of inhibition rate, radicle length, and plumule fresh weight of cucumber test tube plantlets.

PCA of each strain in the combination: PCA of the change value caused by adding the original combination was used to evaluate the contribution degree of each strain to the combination.

### Determination of evaluation index of consortia co-culture filtrate

Determination of the mycelial dry weight: The co-culture medium 10 mL of *Trichoderma* co-cultured for 5 days was filtered on quantitative filter paper by a vacuum pump, washed twice with pure water, and the medium was washed away. Cultures were dried at 105 °C until the culture quality was constant.

Inhibition rate of co-culture filtrate to *F. oxysporum*: The 5-day co-culture of 5 mL *Trichoderma* was mixed with PDA, and then the *F. oxysporum* discs were placed on the centre of the plate. The plates were cultured in a constant temperature incubator at 28 °C for 5 days. After 5 days, the diameter of the pathogen was measured, and the inhibition rate was calculated as follows [41, 42]:

$$ {\text{Inhibition rate}}\left( \% \right) = {{\left( \begin{gathered}  {\text{colony diameter in the control group }} \hfill \\  - {\text{ colony diameter in the treatment group}} \hfill \\ \end{gathered} \right)} \mathord{\left/ {\vphantom {{\left( \begin{gathered}  {\text{colony diameter in the control group }} \hfill \\  - {\text{ colony diameter in the treatment group}} \hfill \\ \end{gathered} \right)} {{\text{colony diameter in the control group}} \times 100.}}} \right. \kern-\nulldelimiterspace} {{\text{colony diameter in the control group}} \times 100.}} $$

The growth-promoting effect of fermentation filtrate on cucumber test tube plantlets was consistent with that of *Trichoderma*.

### Determination of the optimal consortia based on the seed germination assay

The concentration of the co-culture fermentation filtrate was diluted 50 times, 100 times, or 200 times with sterile distilled water, and sterile distilled water was used as a control. The seeds were soaked in 10 mL fermentation filtrate for 24 h, and sterile distilled water was used as a control. Twenty sterilized cucumber seeds of the same size were selected and placed in a 9 cm culture medium with 2 layers of filter paper. Three cucumber seeds per treatment were parallel and placed in a 26 °C artificial climate box. The determination of seed germination and radicle growth index refers to the method of Li et al. [43]. The number of germinated seeds was counted, and the germination rate was calculated every day. In each treatment, twenty cucumber plumules were selected (repeated in 3 groups). The wet cucumber seeds were dried with absorbent paper, the fresh weight was determined, and the total length of the radicle was measured.

$$ {\text{Germination rate}}\left( \% \right) = \frac{{{\text{number of germinated seeds}}}}{{{\text{number of seeds for testing}}}} \times {\text{1}}00. $$

### *Trichoderma* consortia co-culture preparation and bioassay

#### Co-culture process

Fifty litre fermentation tank culture: Four *Trichoderma* strains were separately cultured in PD medium over 2 days as *Trichoderma* starter inoculum. The fermentation medium was filled with 70% of the medium and sterilized at 121 °C for 30 min. A 0.33% *Trichoderma* seed culture was inoculated into the fermentation medium and grown at 28 °C and 200 rpm, and the pH was maintained under natural conditions.

#### Analysis of free amino acids in the co-fermentation filtrate

The sample was vortexed for 1 min and centrifuged at 8000 rpm at 4 °C for 10 min, and the supernatant was obtained [29]. The type and content of free amino acids in the culture supernatant were determined by an automatic amino acid analyser (Hitachi Lmuri 8900).

#### Functional analysis of co-cultures and co-culture filtrate

The four strains were cultured separately and co-cultured to prepare single cultures, single culture filtrate or supernatant, co-cultures, co-cultures filtrate or supernatant. A mixture of single strain monoculture or filtrate and consortia co-culture or filtrate were prepared to determine the separate contribution of each culture.

### Field experiment of cucumber growth promotion

Sterilization and germination of cucumber seeds were carried out with reference to the above method. After 15 days, cucumber seedlings with the same growth vigour were selected and transplanted into a greenhouse located in the experimental field of Shanghai Jiaotong University. On the day of transplanting, the fermentation filtrate was diluted at a ratio of 1:100, and 100 mL of fermentation broth per cucumber was irrigated into the cucumber roots. The treatment was carried out every 10 days. Cucumber growth indicators, such as plant height, stem diameter, chlorophyll, fresh weight, and other indicators, were measured.

### Data analysis

All experiments were studied based on different replications and were repeated at least three times, with reproducible results. The graphs were constructed using Microsoft Office Excel and GraphPad Prism 9 with standard error bars. The results shown are the average of replicates along with the standard error of the mean values. For multiple comparisons, two-way ANOVA with post hoc LSD and Duncan’s test were carried out using SPSS 2.0. Student’s t test was conducted to examine the differences in gene expression using SPSS 2.0. P < 0.05 was considered significant.

## Supplementary Information

**Additional file 1: Table S1.** *Trichoderma* strains used in this study. **Fig. S1** Detection of antagonistic characteristics and functions of different *Trichoderma* strains.

**Additional file 2**. Collection Proof of Strain CM100Z4.

**Additional file 3**. Collection Proof of Strain GDFS1009.

**Additional file 4**. Collection Proof of Strain SBW10264.

**Additional file 5**. Collection Proof of Strain RW10569.

**Additional file 6.** Oral Toxicity Tvaluation of Four Strains *Trichoderma*.

## Data Availability

All data generated during this study are included in this published article.
