# Supplementary material for: Designing synthetic consortia of Trichoderma strains that improve antagonistic activities against pathogens and cucumber seedling growth
Source: Microb Cell Fact. 2022 Nov 11;21:234. doi: 10.1186/s12934-022-01959-2 (PMC9652886; doi:10.1186/s12934-022-01959-2)
Supplement: Supplementary file 1 — Additional file 1: Table S1. Trichoderma strains used in this study. Fig. S1 Detection of antagonistic characteristics and functions of different Trichoderma strains. [file 12934_2022_1959_MOESM1_ESM.docx]

**Additional file 1**

**Table S1 *Trichoderma* strains used in this study**

| Species | Strain number | Strain code | Collecting locations |
| --- | --- | --- | --- |
| *T.atroviride* | CTCCSJ-F-KY10039 | 10039 | Yunnan-Forest-Soil |
|  | CTCCSJ-F-KY10043 | 10043 | Yunnan-Forest-Soil |
|  | CTCCSJ-W-RW10362 | 10362 | Hainan-Wetland-Soil |
|  | CTCCSJ-A-SG3403 | 3403 | Shandong-Filed-Soil |
| *T.harzianum* | CTCCSJ-W-AW10289 | 10289 | Hainan-Wetland-Soil |
|  | CTCCSJ-W-RW10569 | 10569 | Hainan-Wetland-Soil |
| *T.asperellum* | CTCCSJ-W-RW10420 | 10420 | Hainan-Wetland-Soil |
|  | CTCCSJ-W-AW-10440 | 10440 | Hainan-Wetland-Soil |
|  | CTCCSJ-W-RW10539 | 10539 | Hainan-Wetland-Soil |
|  | CTCCSJ-A-GDFS1009 | 1009 | Guangdong-Filed-Soil |
|  | CTCCSJ-W-SBW10264 | 10264 | Hainan-Wetland-Soil |
| *T.hamatum* | CTCCSJ-A-YM12048 | 12048 | Dandong-Filed-Root |
| *T.aureoviride* | CTCCSJ-W-RW10644 | 10644 | Hainan-Wetland-Soil |
| *T.viride* | CTCCSJ-A-YM12057 | 12057 | Dandong-Filed-Root |
| *T.asperelloides* | CTCCSJ-A-CM100Z4 | Z4-1 | Shanghai-Chongming-citrus peel |


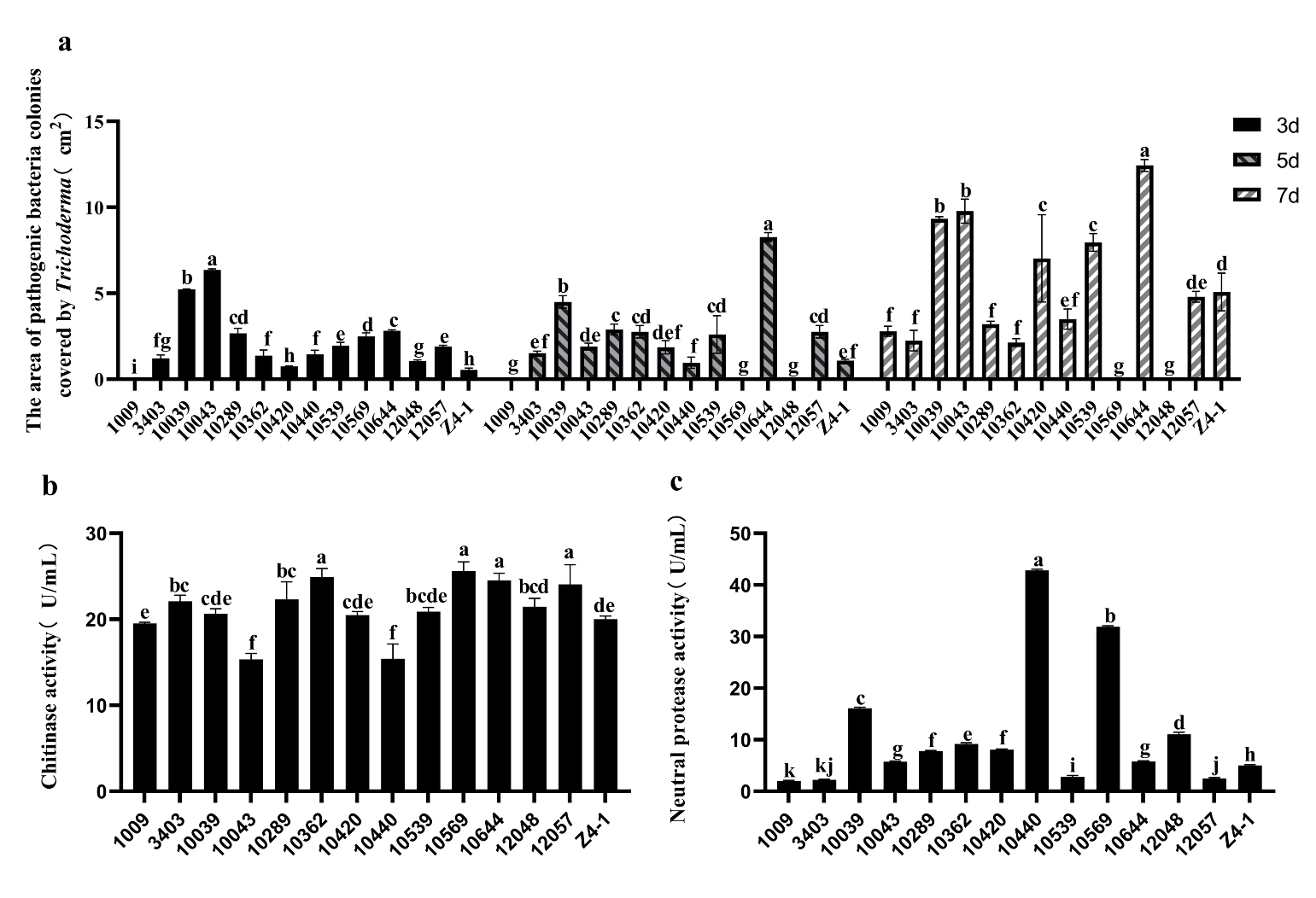


**Fig. S1** Detection of antagonistic characteristics and functions of different *Trichoderma*. **a** Antagonistic coverage area of Trichoderma against *F. oxysporum*. **b** Chitinase activity assay. **c** Neutral protease activity assay. The results are the means of 3 replicates for each treatment; the value is the standard error of the mean. Different letters above the bars indicate significant differences (P < 0.05) based on ANOVA.
