# Supplementary figures and images for: Designing synthetic consortia of Trichoderma strains that improve antagonistic activities against pathogens and cucumber seedling growth
Source: Microb Cell Fact. 2022 Nov 11;21:234. doi: 10.1186/s12934-022-01959-2 (PMC9652886; doi:10.1186/s12934-022-01959-2)

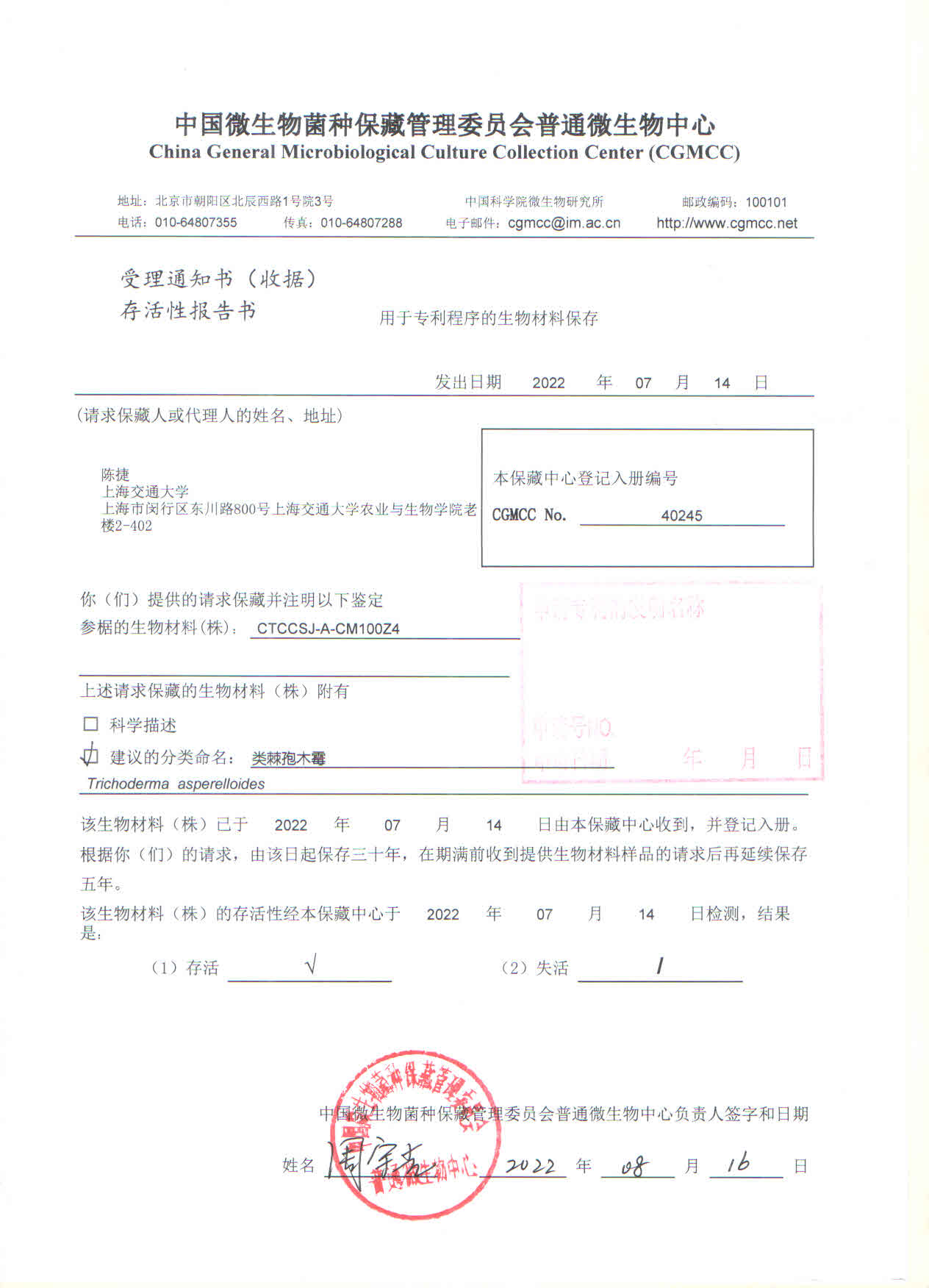

Supplement: Supplementary file 2 — Additional file 2. Collection Proof of Strain CM100Z4. [file 12934_2022_1959_MOESM2_ESM.jpg]

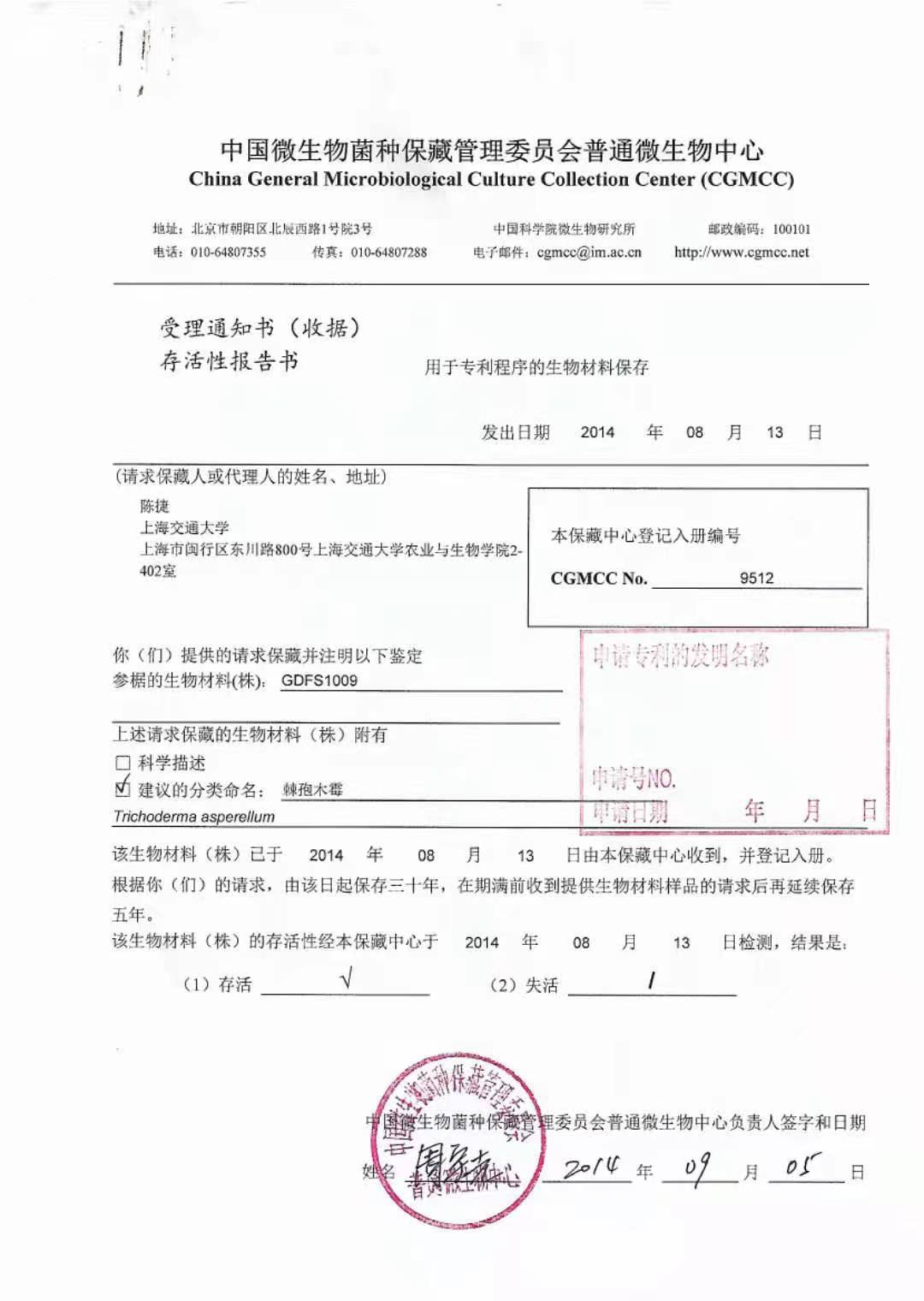

Supplement: Supplementary file 3 — Additional file 3. Collection Proof of Strain GDFS1009. [file 12934_2022_1959_MOESM3_ESM.jpg]

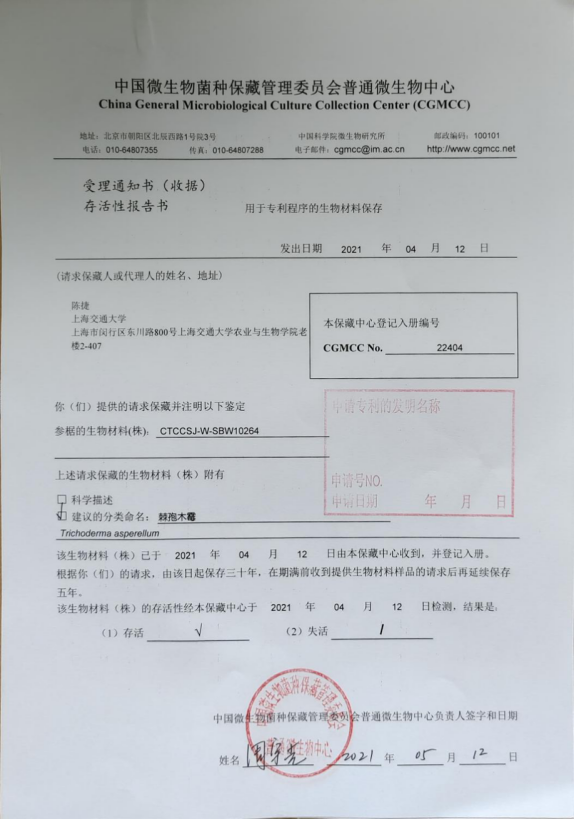

Supplement: Supplementary file 4 — Additional file 4. Collection Proof of Strain SBW10264. [file 12934_2022_1959_MOESM4_ESM.jpg]

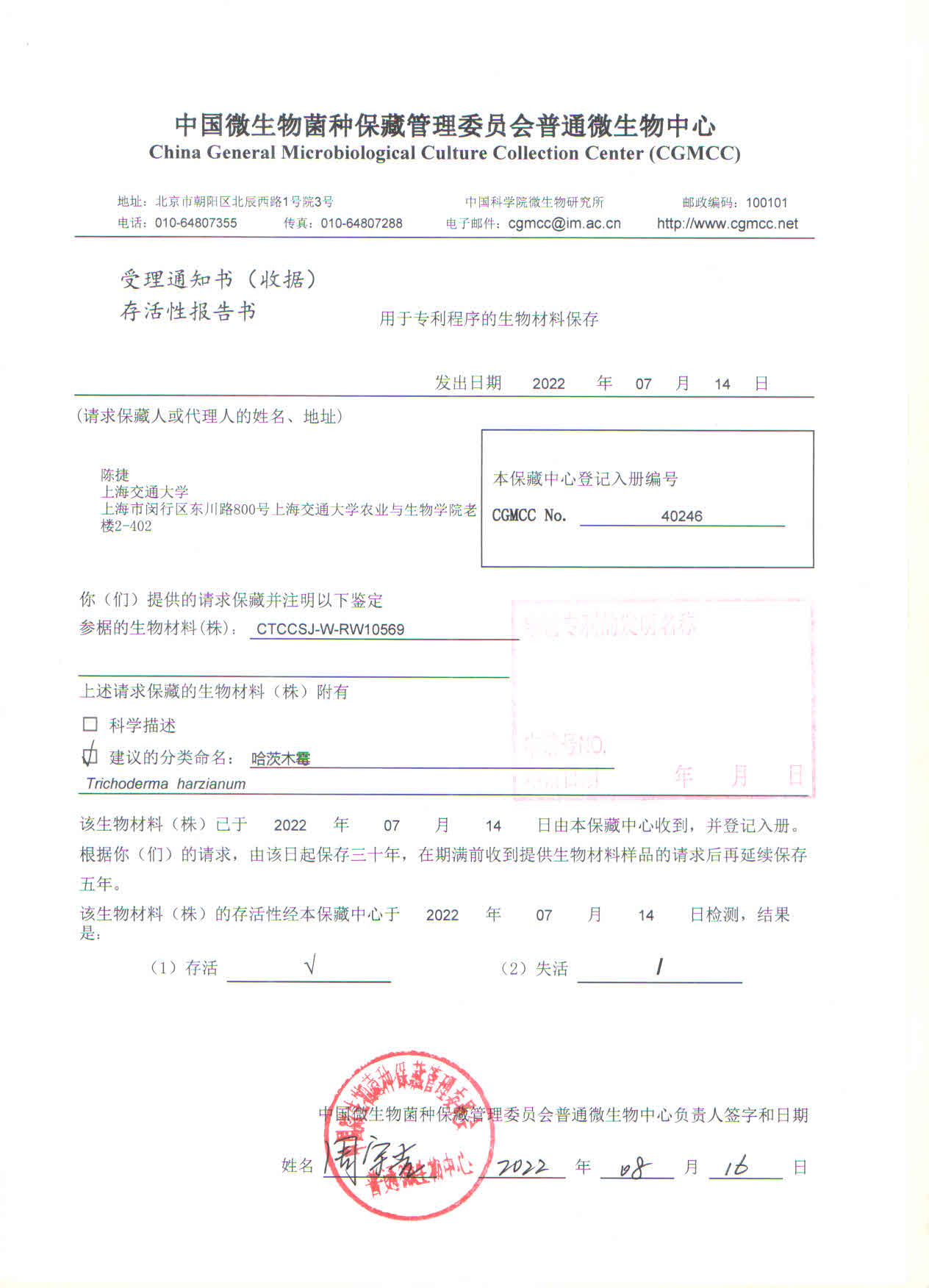

Supplement: Supplementary file 5 — Additional file 5. Collection Proof of Strain RW10569. [file 12934_2022_1959_MOESM5_ESM.jpg]
