# Supplementary material for: Designing synthetic consortia of Trichoderma strains that improve antagonistic activities against pathogens and cucumber seedling growth
Source: Microb Cell Fact. 2022 Nov 11;21:234. doi: 10.1186/s12934-022-01959-2 (PMC9652886; doi:10.1186/s12934-022-01959-2)
Supplement: Supplementary file 6 — Additional file 6. Oral Toxicity Tvaluation of Four Strains Trichoderma. [file 12934_2022_1959_MOESM6_ESM.pdf]

复达检测集团
FUDA ANALYTICAL TESTING GROUP

测 试 报 告

样品名称 株木霉共发酵产物

委托单位 上海交通大学

报告编号 FT-20220715070

上海复达检测技术集团有限公司

中国▪上海 上海市杨浦区复旦大学复华楼二楼（国权路 525 号）
服务热线：021-61996230 E-mail:fudan.edu@fudanfuxin.com

样品名称	株木霉共发酵产物		
样品数量	1	样品批号	/
样品状态	完好	样品编号	FT220715070
委托单位	上海交通大学		
委托单位通讯资料	上海市闵行区东川路 800 号上海交通大学农业与生物学院		
测试类别	委托测试		
到样日期	2022 年 07 月 20 日		
测试周期	2022 年 07 月 20 日 — 2022 年 08 月 31 日		
测试依据和方法	详见本报告测试结果汇总页。		
测试结果	本报告仅提供实测值。详见本报告测试结果汇总页。		
备注	——		

编制：________　　签发：________

审核：________　　日期：2022-09-05

急性经口毒性试验

1. 材料和方法

1.1. 受试样品处理

称取 10.0760g 样品置于烧杯中取少量纯水将样品混匀后倒入 20mL 容量瓶内，以少量纯水多次冲洗烧杯一并倒入容量瓶中，加入纯水定容至刻度线，充分摇匀后装入样品管，贴上标签备用，样品现配现用。

灌胃体积：2.0mL/100g·BW

1.2. 溶剂：纯水；

1.3. 实验动物和饲养环境

1.3.1. 实验动物信息

种属：小鼠；

品系：ICR；

级别：SPF 级；

动物数量：20 只；

动物性别：雌雄各半，雌性动物未孕和未曾产仔；

周龄：5 周龄；

体重：18.0g～21.6g，同性别体重不超过均值的±20%；

1.3.2. 饲养环境信息

设施：屏障环境；

温度：22.1℃～24.0℃；

相对湿度：49.6%～59.3%；

1.3.3. 饲料信息

饲料名称：Co60 辐照鼠料；

生产日期：2022.05.28；

保质期：6 个月；

批号：22050118；

1.3.4. 动物饮用水信息

一级 RO 超滤水（加入次氯酸钠，将水中游离氯含量控制在 2-3ppm 除菌）。经饮水嘴直接供动物自由饮用。

1.4. 试验方法

试验前动物在屏障环境动物房中适应 5 天，染毒前动物禁食 4 小时。试验开始后，采用最大限量法，灌胃剂量为 10076.0mg/kg。染毒后继续禁食 1 小时，每天观察中毒症状或行为变化，每周称重一次。观察并记录染毒过程和观察期内动物的中毒和死亡情况，观察周期 14 天，观察期结束后，处死存活动物并进行大体解剖。

2. 试验结果

实验动物在染毒 14 天内未见任何中毒症状和中毒死亡；雌雄动物的体重未见异常。实验观察结束，对受试动物进行大体解剖检查也未见异常变化。LD50＞10076.0mg/kg。

在本试验条件下，受试样品对 ICR 小鼠的急性经口 LD50＞5000mg/kg。根据急性毒性（LD50）剂量分级，该样品属实际无毒级。

性别	动物数（只）	体重（$\bar{x}$±SD）（g）				死亡数（只）	死亡率（%）
		0 天	7 天	14 天	14 天增重		
雄性	10	20.1±1.17	25.3±1.18	29.4±1.76	9.3±1.01	0	0
雌性	10	20.1±1.13	23.6±1.13	26.9±1.74	6.8±0.82	0	0

报告结束

注 意 事 项

1、本报告无“上海复达检测技术集团有限公司报告专用章”无效。

2、未经本单位书面批准，不得自行复制本报告。如确有需要，应持公函或介绍信申请复制。

3、对本报告若有异议，应于收到本报告之日起 10 日内向本实验室提出，过期不予受理。

4、报告仅对来样负责，测试结束后样品原则上保留时间为 30 日 。

5、本机构对委托单位技术文件、报告文本、合同文件等商业秘密履行保密义务。

6、报告未加盖资质认定标志（CMA）时，数据和结果仅作为科研、教学和内部质量控制用，不作为社会公正性数据。中英文报告内容以中文为准。

中国▪上海 上海市杨浦区复旦大学复华楼二楼（国权路 525 号）
服务热线：021-61996230 E-mail:fudan.edu@fudanfuxin.com
